# Effect of novel training to normalize altered finger force direction post-stroke: study protocol for a double-blind randomized controlled trial

**DOI:** 10.1186/s13063-022-06224-w

**Published:** 2022-04-12

**Authors:** Na Jin Seo, Derek G. Kamper, Viswanathan Ramakrishnan, Jillian B. Harvey, Christian Finetto, Christian Schranz, Gabrielle Scronce, Kristen Coupland, Keith Howard, Jenna Blaschke, Adam Baker, Caitlyn Meinzer, Craig A. Velozo, Robert J. Adams

**Affiliations:** 1 Ralph H. Johnson VA Medical Center, Charleston, SC USA; 2 Department of Rehabilitation Sciences, Medical University of South Carolina, 151B Rutledge Ave, MSC 962, Charleston, SC 29425 USA; 3 Department of Health Science and Research, Medical University of South Carolina, 77 President St., MSC 700, Charleston, SC 29425 USA; 4 Joint Department of Biomedical Engineering, North Carolina State University, University of North Carolina at Chapel Hill, Chapel Hill, NC Raleigh, USA; 5 Department of Public Health Sciences, Medical University of South Carolina, 135 Cannon St, Charleston, SC 29425 USA; 6 Department of Healthcare Leadership and Management, Medical University of South Carolina, 151B Rutledge Ave, MSC 962, Charleston, SC 29425 USA; 7 Department of Health Sciences and Research, Medical University of South Carolina, 77 President St, MSC 700, Charleston, SC 29425 USA; 8 Division of Occupational Therapy, Department of Rehabilitation Sciences, Medical University of South Carolina, 151B Rutledge Ave, MSC 962, Charleston, SC 29425 USA; 9 Department of Neurology, Medical University of South Carolina, 96 Jonathan Lucas St, MSC 606, Charleston, SC 29425 USA

**Keywords:** Stroke, Upper extremity, Physical rehabilitation, Hand function, Grip force, EMG, Biofeedback, Control, Paralysis, Randomized controlled trial

## Abstract

**Background:**

Functional task performance requires proper control of both movement and force generation in three-dimensional space, especially for the hand. Control of force in three dimensions, however, is not explicitly treated in current physical rehabilitation. To address this gap in treatment, we have developed a tool to provide visual feedback on three-dimensional finger force. Our objective is to examine the effectiveness of training with this tool to restore hand function in stroke survivors.

**Methods:**

Double-blind randomized controlled trial. All participants undergo 18 1-h training sessions to practice generating volitional finger force of various target directions and magnitudes. The experimental group receives feedback on both force direction and magnitude, while the control group receives feedback on force magnitude only. The primary outcome is hand function as measured by the Action Research Arm Test. Other outcomes include the Box and Block Test, Stroke Impact Scale, ability to direct finger force, muscle activation pattern, and qualitative interviews.

**Discussion:**

The protocol for this clinical trial is described in detail. The results of this study will reveal whether explicit training of finger force direction in stroke survivors leads to improved motor control of the hand. This study will also improve the understanding of neuromuscular mechanisms underlying the recovery of hand function.

**Trial registration:**

ClinicalTrials.govNCT03995069. Registered on June 21, 2019

**Supplementary Information:**

The online version contains supplementary material available at 10.1186/s13063-022-06224-w.

## Administrative information

Note: The numbers in curly brackets in this protocol refer to SPIRIT checklist item numbers. The order of the items has been modified to group similar items (see http://www.equator-network.org/reporting-guidelines/spirit-2013-statement-defining-standard-protocol-items-for-clinical-trials/).

**Table:** 

Title {1}	Effect of novel training to normalize altered finger force direction post-stroke: study protocol for a double-blind randomized controlled trial
Trial registration {2a and 2b}.	NCT03995069. Clinicaltrials.gov
Protocol version {3}	#9. 2/8/2022
Funding {4}	VA RR&D Merit 1I01RX003066-01A1
Author details {5a}	Na Jin Seo, PhD, Ralph H. Johnson VA Medical Center, Charleston, SC, USA. Department of Rehabilitation Sciences, Department of Health Science and Research, Medical University of South Carolina, Charleston, SC, USA. ORCID 0000-0001-6446-5905. NaJin.Seo@va.gov, seon@musc.edu. 151B Rutledge Ave, MSC 962, Charleston, SC 29425. 843-792-0084. Derek G. Kamper, PhD, Joint Department of Biomedical Engineering, North Carolina State University, University of North Carolina at Chapel Hill, Raleigh/Chapel Hill, NC, USA. ORCID 0000-0001-9048-4106. dgkamper@ncsu.edu. 4402C Engineering Building III, Raleigh, NC 27695. 919-515-4411. Viswanathan Ramakrishnan, PhD, Department of Public Health Sciences, Medical University of South Carolina, Charleston, SC, USA. ORCID 0000-0002-4098-0539. ramakris@musc.edu. 135 Cannon St, Charleston, SC 29425. 843-876-1153. Jillian B. Harvey, PhD, Department of Healthcare Leadership and Management, Medical University of South Carolina, Charleston, SC, USA. ORCID 0000-0002-6814-8226. harveyji@musc.edu. 151B Rutledge Ave, MSC 962, Charleston, SC 29425. 843-792-3431. Christian Finetto, PhD, Department of Health Sciences and Research, Medical University of South Carolina, Charleston, SC, USA. ORCID 0000-0003-0520-2034. finetto@musc.edu. 77 President St, MSC 700, Charleston, SC 29425. 843-792-5645. Christian Schranz, PhD, Department of Health Sciences and Research, Medical University of South Carolina, Charleston, SC, USA. ORCID: 0000-0003-1102-7180. schranz@musc.edu. 77 President St, MSC 700, Charleston, SC 29425. Gabrielle Scronce, DPT, PhD, Department of Health Sciences and Research, Medical University of South Carolina, Charleston, SC, USA. ORCID 0000-0002-3861-1371. scronce@musc.edu. 77 President St, MSC 700, Charleston, SC 29425. Kristen Coupland, MS, OTR/L, CSRS, CDRS, Department of Health Sciences and Research, Medical University of South Carolina, Charleston, SC, USA. coupland@musc.edu. 77 President St, MSC 700, Charleston, SC 29425. Keith Howard, Department of Health Sciences and Research, Medical University of South Carolina, Charleston, SC, USA. howardke@musc.edu. 77 President St, MSC 700, Charleston, SC 29425. 843-792-2917. Jenna Blaschke, BA, Division of Occupational Therapy, Department of Rehabilitation Sciences, Medical University of South Carolina, Charleston, SC, USA. blaschkj@musc.edu. 151B Rutledge Ave, MSC 962, Charleston, SC 29425. 803-459-6403. Adam Baker, BS, Department of Health Sciences and Research, Medical University of South Carolina, Charleston, SC, USA. bakerdon@musc.edu. 77 President St., MSC 700, Charleston, SC 29425 Caitlyn Meinzer, PhD, Department of Public Health Sciences, Medical University of South Carolina, Charleston, SC, USA. ellerbcn@musc.edu. 135 Cannon St, Charleston, SC 29425. 843-792-6588. Craig A. Velozo, PhD, Division of Occupational Therapy, Department of Rehabilitation Sciences, Medical University of South Carolina, Charleston, SC, USA. velozo@musc.edu. 151B Rutledge Ave, MSC 962, Charleston, SC 29425. 803-459-6403. Robert J. Adams, MD, Department of Neurology, Medical University of South Carolina, Charleston, SC, USA. adamsrj@musc.edu. 96 Jonathan Lucas St, MSC 606, Charleston, SC 29425. 843-792-3020.
Name and contact information for the trial sponsor {5b}	VA Office of Research and Development. Ralph H. Johnson VA Medical Center, 109 Bee St., Charleston, SC, 29401.
Role of sponsor {5c}	Oversight of regulatory requirements.

## Introduction

### Background and rationale {6a}

Our hands constitute our primary means of interacting with the external world. They allow us to complete activities of daily living and dexterously manipulate objects such as tools, dishes, and smartphones. Unfortunately, more than two-thirds of stroke survivors have hand impairment [1]. Hand impairment results in objects being mishandled or not handled at all and failure at task execution. Thus, hand function, and thereby utility of the entire upper extremity, can be dramatically diminished, leading to decreased independence, diminished work opportunities, and reduced satisfaction from leisure pursuits [2].

Successful object manipulation requires not only proper finger placement and movement, but also digit *force* control. Specifically, fingertip and thumb tip forces need to be properly directed and scaled to accomplish tasks (e.g., securely holding a paper cup against gravity, without crushing the cup, while coffee is poured into it) [3–9]. This force generation must be controlled dynamically as the object properties change or the finger posture changes [10–14]. After stroke, not only finger movement [15–17] but also force control [18] is impaired. Stroke survivors exhibit impairment in the ability to apply force in the proper direction per task demand [19, 20]. The altered grip force directions result in difficulty in picking up objects for activities of daily living as the objects slip out of the hand [21].

Neuromechanical investigations revealed that this altered paretic grip force direction arose from altered muscle activation patterns [19, 20]. Investigators have suggested that the central nervous system uses two separate neural strategies to control digit force and movement [22]. Therefore, both neural strategies must be *independently* rehabilitated to achieve proper hand function [23]. Current therapy focuses on the training of volitional movement which inherently provides visual feedback for both the client and the therapist [24]. Unfortunately, digit *force* control is rarely explicitly addressed in therapy. Currently, the only explicit visual feedback of force direction occurs when an object grasped by the paretic fingers is dropped or cannot be held safely. While skin mechanoreceptors can provide sensory information about contact forces with objects [25–27], net three-dimensional force direction may be difficult to explicitly recognize, and this sensation may be compromised in stroke survivors [28, 29]. Treatment of force generation can be much more challenging for therapists to direct as they receive only gross cues about client performance and have few tools for guiding repetitive practice.

To fill this gap in treatment, we have developed a novel training tool in which the three-dimensional (3D) grip force applied to an instrumented object is displayed in real time on a computer screen. This training tool enables stroke survivors to practice generating fingertip force in a variety of magnitudes and directions. A pilot study using a single group pre-post design showed the following preliminary evidence: (1) stroke survivors improved their control of digit force direction using the tool, (2) the improvement was accompanied by changes in muscle activation pattern, and (3) the improved force direction translated to an increased ability to manipulate objects [30].

As a next step, a randomized controlled trial is described herein. This research is expected to fill the gap in the current rehabilitation therapy treatment by evaluating a tool to enable training of digit force control with explicit feedback. This training tool for force control will complement the existing movement training, both of which are independently essential to obtain proper hand function [22].

### Objectives {7}

The objective of this study is to determine if training force directional control with this tool is effective in restoring hand function post-stroke. Specifically, we will determine the effect of this training on clinical hand function, the ability to control digit force direction, and muscle activation patterns.

### Trial design {8}

The trial design is a double-blind randomized controlled trial involving two parallel groups. Participants and outcome assessors will be blinded to the group assignment to increase the rigor of the study. Participants will be randomly assigned to either the experimental or control group (see the “Assignment of interventions: allocation” section). Half will be in the experimental group, and the other half will be in the control group. Both groups will undergo training to practice generating volitional finger force to various target directions and magnitudes. The experimental group will receive feedback on both force direction and magnitude. The control group will receive feedback on force magnitude in the target direction only, which represents the current force feedback capacity. The superiority of the experimental intervention over the control will be examined.

## Methods: participants, interventions, and outcomes

### Study setting {9}

The study setting is the research laboratory of the Ralph J. Johnson Veterans Affairs (VA) Medical Center, Charleston, SC, USA.

### Eligibility criteria {10}

#### Inclusion criteria

The following are the inclusion criteria:

- Survived a stroke at least 3 months ago
- Moderate to severe hand impairment (Chedoke-McMaster [31] Hand Stage 2–4)
- Ability to generate palpable volitional grip force upon cue
- Sufficient cognitive ability to participate (NIH Stroke Scale [32], questions and commands score = 0–1)
- Ability to recognize all quadrants of the visual field (NIH Stroke Scale, visual field test score = 0)

#### Exclusion criteria

The following are the exclusion criteria:

- Concurrent upper limb rehabilitation
- Inability to follow 2-step commands
- Severe muscle tone prohibiting the passive movement of the fingers or proper placement of the fingers on the force sensors as needed to participate in the training (Modified Ashworth Scale [33] = 4–5 out of 5)
- Change in spasticity medication or botulinum toxin injection in the upper limb within 3 months prior to or during enrollment
- Total sensory loss on fingertips (NIH Stroke Scale [32], sensory score = 2)
- Comorbidity (e.g., orthopedic conditions that limit ranges of motion, premorbid neurologic conditions)
- Language barrier or cognitive impairment that precludes providing consent

### Who will take informed consent? {26a}

Research staff approved by the Medical University of South Carolina Institutional Review Board will take the informed consent. The consent process will take place in a private room when the potential participant comes to the laboratory at a scheduled time agreed upon between the study personnel and the participant. The content of the consent will be verbally explained to the participant, and the participant will be asked to raise any questions and concerns.

### Additional consent provisions for collection and use of participant data and biological specimens {26b}

The consent includes sharing of de-identified data with the public and other investigators in publications and ClinicalTrials.gov. The consent also includes sharing of de-identified data with the collaborating site, North Carolina State University, for analysis. In addition, authorization for use and release of individually identifiable health information collected for research with the Institutional Review Board of the Medical University of South Carolina, the funding agency, the collaborating sites of Medical University of South Carolina, and VA Centralized Transcription Services Program will be obtained in writing. This trial does not involve collecting biological specimens for storage.

### Interventions

#### Explanation for the choice of comparators {6b}

The control group receives feedback on the force magnitude in the target direction only and receives no explicit feedback on forces in other directions and, thus, no training in force directional control. The control condition is akin to a standard treatment of repeatedly squeezing a ball. The control condition would be more accessible in a clinic setting. Specifically, force magnitude feedback can be provided with a conventional grip dynamometer readily available in rehabilitation clinics, whereas feedback of both force magnitude and direction requires multi-axial force sensors with specialized software which tend to be more expensive. While the control condition is more accessible [34], it does not provide explicit feedback for digit force directional control which is hypothesized to have a significant role in improving hand force control and manipulation ability. The control condition may be viewed as analogous to practicing multi-joint upper limb movement within the abnormal flexion synergy pattern [35], without explicit feedback to break out of the abnormal flexion synergy pattern. The experimental vs. control comparison will determine the therapeutic value of the force direction feedback.

#### Intervention description {11a}

##### Intervention duration

All participants will complete a 1-h force training session, 3 times per week for 6 weeks (a total of 18 training sessions). This schedule simulates the outpatient rehabilitation model [36] and thus facilitates the potential translation of the protocol for implementation in rehabilitation practice.

##### Force training

**Fig. 1:**
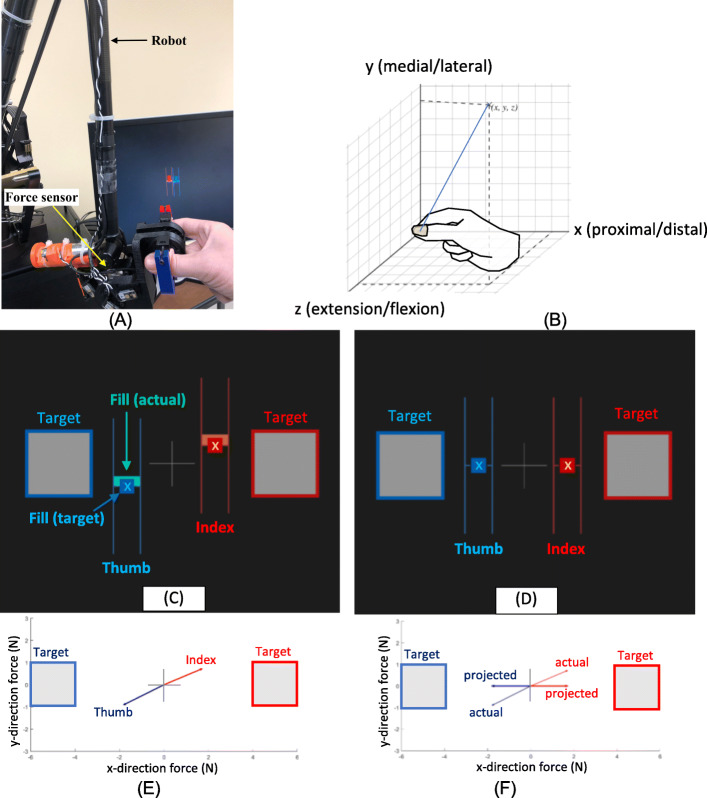
**A** Finger apparatus attached to the robot. **B** Notation system for describing target directions in ***x***, ***y***, and ***z*** axes. An example of visual feedback is shown for the experimental (**C**) and control (**D**) groups. Thumb force is shown as a blue ladder (composed of the two blue parallel lines and X in the center). Finger force is shown as a red ladder. The location for the center of the ladder (X) is determined by the digit force in the ***x*** and ***y*** direction, relative to the zero (the cross in the center). Digit force in the ***z*** direction is shown as the “fill” of the ladder. The goal is to place the center of the ladder inside the target, while matching the fill to the fill target. The actual force in all 3 directions is shown in the visual feedback to the experimental group (**C**). The actual force vector in the ***x*** and ***y*** directions is shown for illustration purposes only in **E**. For the control group, the same actual force is projected to the target direction in 3D (**F**, showing only 2 axes for illustration only). The projected force is shown for visual feedback for the control group (**D**)

For those whose hands can be comfortably placed in our instrument in the precision pinch posture [37], training will take place in a precision pinch posture. Otherwise, a cylindrical grip posture will be used as described later. For precision pinch, the intervention primarily focuses on the training of isometric force production with the index finger and thumb. Participants place their finger and thumb into a custom apparatus that includes two 6-axis force sensors (Mini45, ATI Industrial Automation Inc., Apex, NC, USA) (Fig. 1A). The apparatus is attached to a robot (Phantom® Premium™ 3.0/6DOF, 3D Systems Inc.) that provides stability to achieve the target force generation.

A total of 14 target force directions will be trained. The target directions for the thumb are (0, 0, ± *x*), (0, ± *y*, 0), (0, 0, ± *z*), and (± *x*, ± *y*, ± *z*), with ± *x*, + *y*, and + *z* representing proximal/distal, medial/lateral, and extension/flexion direction of the thumb, respectively (Fig. 1B). These training directions encompass the whole sphere from the digit tip. The corresponding target directions for the finger are the opposite of that for the thumb.

A custom-developed computer program for the training provides visual feedback for performance relative to the targets. An example of visual feedback on the computer screen is shown for the experimental group (Fig. 1C) and for the control group (Fig. 1D). Thumb force is shown as a blue ladder (composed of the two blue parallel lines and X in the center). Finger force is shown as a red ladder. For the treatment group, the *x* and *y* locations for the center of the ladder (marked as X in Fig. 1C) are determined by the digit force in the *x* and *y* directions (proximal/distal and medial/lateral direction as shown in Fig. 1B). The cross in the center (in Fig. 1C) represents zero force in both *x* and *y* directions. The fill of the ladder in the positive or negative direction (from X in Fig. 1C) is determined by the digit force in the *z* direction (gripping/opening direction as shown in Fig. 1B). The goal is to place the center of the ladder inside the target, while matching the fill to the fill target. Specifically, to achieve the example target in Fig. 1C with the right hand, the thumb should produce 5 N in the distal direction (− *x* in Fig. 1B) and the index finger should produce 5 N in the proximal direction (+ *x* in Fig. 1B), while maintaining little force in the *y* and *z* directions. This target mimics sliding the thumb and a finger in the opposite direction to open a plastic produce bag. The experimental group receives the visual feedback of their true force control (Fig. 1E showing 2 axes of the actual force for illustration purposes only). For the control group, the same actual force vector is projected on the target vector in 3D (Fig. 1F showing projection on 2 axes for illustration purposes only). This projected force is shown as visual feedback to the participant, such that force in the non-target direction (e.g., medial/lateral and flexion/extension direction in this example) is shown as zero (Fig. 1D), when it is not zero in reality.

##### Training structure

The 18 training sessions will be structured such that all participants in both groups will experience training with different arm postures, whole-body postures, digits, laterality, force variability, magnitude, and visual feedback conditions in a progressive manner (Table 1). This progression framework enables the variable practice structure (vs. constant practice) that has been shown to induce greater retention in motor learning [38]. The control group will experience the same progression, except that they will receive feedback in force magnitude only.

**Table 1:** Overall structure for the 18 training sessions

Arm posture #	Whole body	Digit	Laterality	Variability	Magnitude	Feedback	No. of sessions
1–6	Sitting	2	Unilateral	65–40%	4–11 N	30 and 0.5 Hz	6 for each arm posture
1, 5, 6	Standing	2	Unilateral	55–40%	4–11 N	30 and 0.5 Hz	3 for each arm posture
1, 6	Walking	2	Unilateral	55–40%	4–11 N	30 and 0.5 Hz	2 for each arm posture
1, 4, 5, 6	Sitting	2	Bimanual fixed	55–40%	5–7 N	30 Hz	1
1, 4, 5, 6	Sitting	2	Bimanual unconstrained	55–40%	5–7 N	30 and 0.5 Hz	1
1, 4, 5, 6	Standing	2	Bimanual fixed	55–40%	5–7 N	30 Hz	1
1, 4, 5, 6	Standing	2	Bimanual unconstrained	55–40%	5–7 N	30 and 0.5 Hz	1
1, 4	Sitting	3	Unilateral	55–40%	5–8 N	30 and 0.5 Hz	1
5, 6	Sitting	3	Unilateral	55–40%	5–8 N	30 and 0.5 Hz	1
1, 4, 5, 6	Sitting	4	Unilateral	55–40%	5–8 N	30 and 0.5 Hz	1

Total 18 sessions

**Fig. 2:**
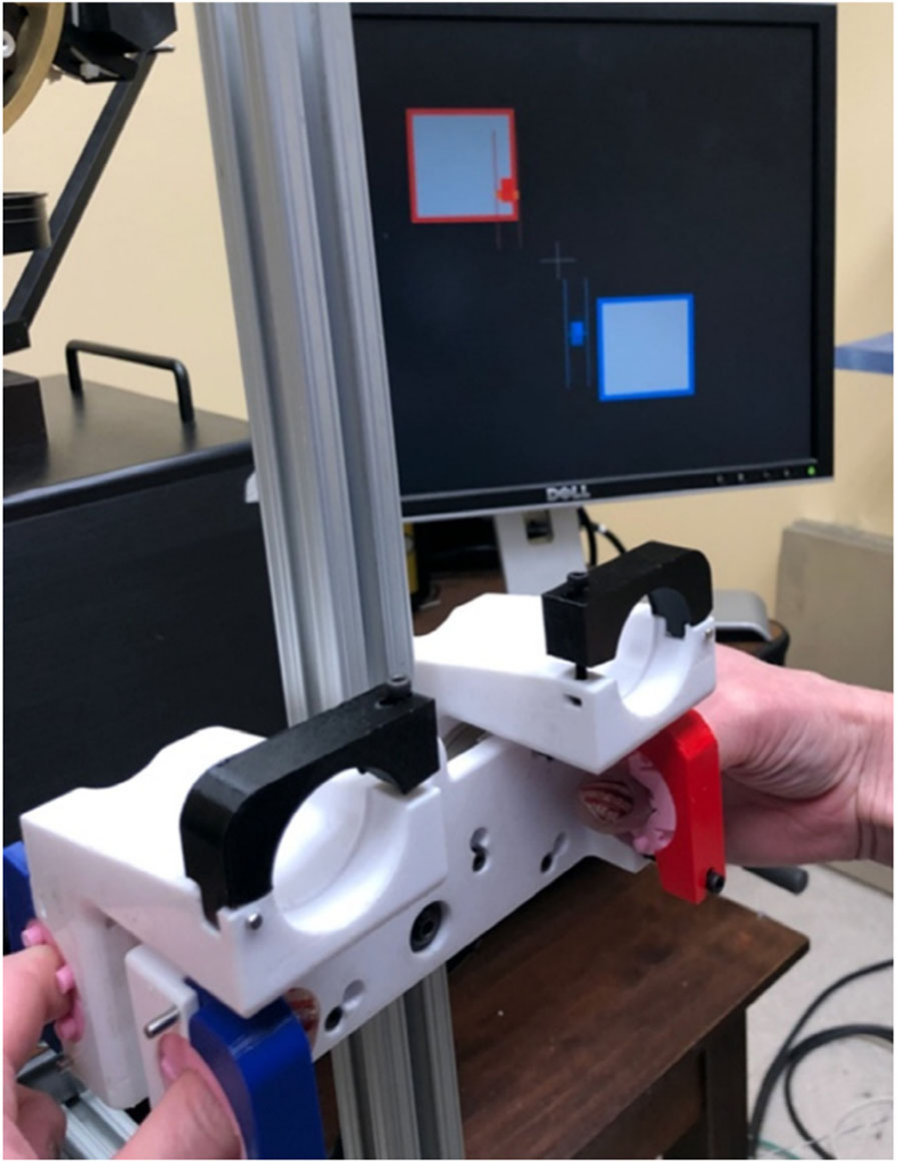
Bilateral training apparatus

Specifically, the following conditions will be employed: upper arm on the side of the body, elbow flexed, and forearm horizontally rested on a forearm rest in (1) midprone, (2) pronation, and (3) supination and forearm midprone with (4) elbow extended, (5) shoulder flexion with an external arm support provided from Armeo Boom (Hocoma AG, Switzerland), and (6) shoulder flexion without support. The whole-body postures will include sitting, standing, and walking on a treadmill (with a harness for safety). These varying arm and whole-body postures will be used because these attributes are known to elicit different levels of abnormal flexion synergy often present in stroke survivors [35] that can impede hand motor control [35, 39–44]. The training will progress by working against the abnormal flexion synergy. The digit used in training will primarily be the 2nd digit (against the thumb), while the 3rd and 4th digits will also be used (Table 1). The laterality condition will include unilateral (i.e., paretic only, Fig. 1A) and bilateral (Fig. 2) as described below.

For the bilateral training, a custom-developed bilateral apparatus with one force sensor for each hand will be used, with visual feedback showing each hand’s index finger forces on a computer screen (Fig. 2). The bilateral apparatus will be fixed for the initial session and unconstrained in the next session to increase the challenge associated with manipulating a stable vs. unconstrained object [45]. For the fixed condition, targets for the two hands will be the same at first (i.e., both hands try to do the same thing, mirroring activity). Later, to dissociate activities between the two hands [46], targets for the two hands will be opposite of each other. For example, targets will require generating opening force with the paretic hand while creating gripping force with the nonparetic hand and vice versa, pull/push mimicking pulling/pushing a cap on a pen, and twist in the opposite directions (mimicking twist off of a cap). For the unconstrained condition, targets will be chosen such that force equilibrium is maintained.

Additional training variables of force variability, magnitude, and visual feedback delay will be modified to adjust difficulty from one set to the next within a training session (Table 1). For example, force variability will be gradually restricted as the participant progresses. Initially, large variability (margin of error) from the target will be allowed. Then, allowed variability will be progressively reduced. The allowed variability will be shown as the size of the square target for vertical and horizontal forces and the thickness of the mark in the ladder for the gripping/opening direction force (Fig. 1C, D). Participants will be required to keep the force vector within the margin of error for 1 s continuously for a successful trial [30]. Furthermore, the target force magnitude will be gradually increased over a range needed to open a conventional bottle, not exceeding approximately half the fingertip strength [47, 48]. Lastly, visual feedback delay will be initially imperceptible (i.e., refresh rate of 30 Hz) and later introduced at a 0.5-Hz refresh rate to solicit increased reliance on intrinsic sensory feedback (vs. external visual feedback).

##### Difficulty adjustment

To keep participants engaged and challenged, the difficulty level of the training will be adjusted per the Challenge Point Framework [49]. Specifically, if the success rate in a single set encompassing all target force directions is > 80%, then one of the difficulty attributes (e.g., force variability, magnitude, visual feedback delay) will change to the next difficulty level for the next set. If the success rate is < 40%, an easier setting will be used for the next set. For the success rates in between, the difficulty level will be maintained in the next set. If the performance plateaus over 3 sets, the next difficulty set will be used to reduce frustration and ensure exposure to various training conditions. A maximum of 30 s will be allowed for each target as in the preliminary study [30] except when explaining the training to the participant in which case 60 s will be allowed.

##### Alternative strategies

**Fig. 3:**
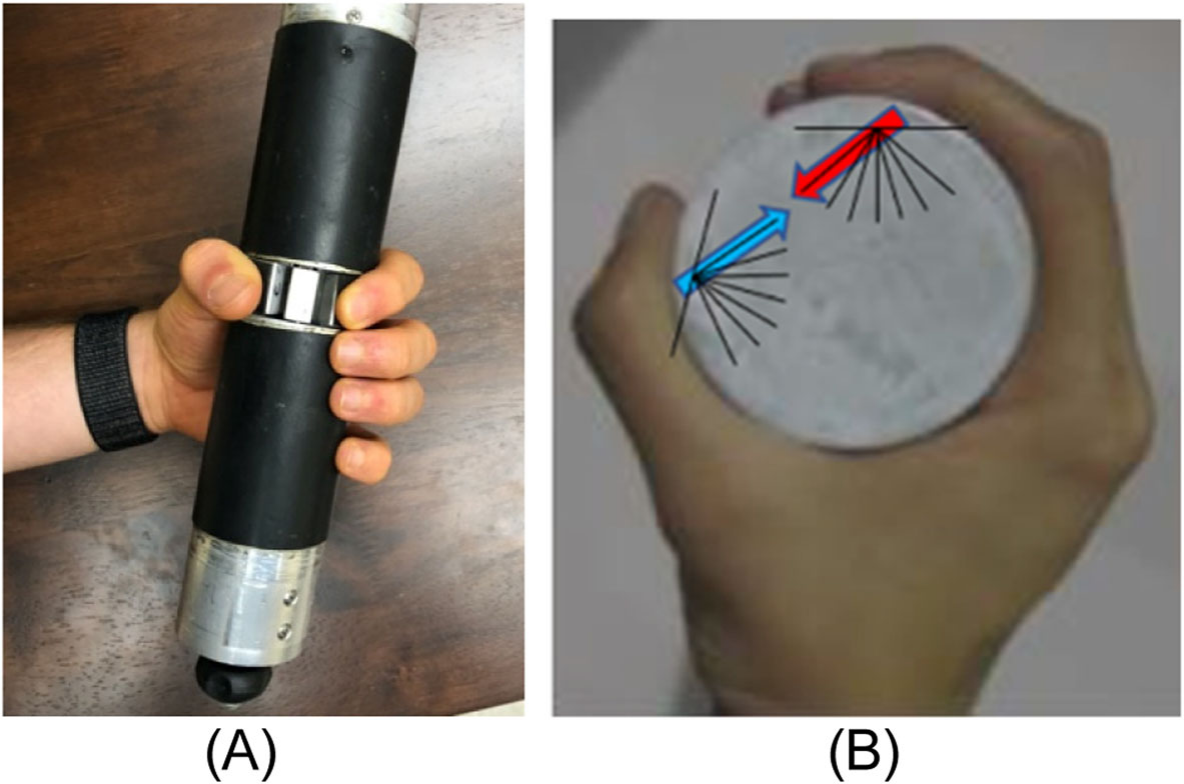
**A** Cylindrical grip device. **B** Force target directions for the cylindrical grip

For those who cannot train in the precision pinch posture, the cylindrical grip posture may be used. We will use a custom-developed cylindrical grip device [20] that can measure multi-axial force for the distal phalanx of the thumb and the finger separately and simultaneously (Fig. 3A). The force directions appropriate for the cylindrical grip will be trained (Fig. 3B). The visual feedback and the progression through various arm postures, whole-body posture, force variability, magnitude, and visual feedback delay conditions will stay the same.

#### Criteria for discontinuing or modifying allocated interventions {11b}

Depending on their functional level, some participants may not be able to perform the training in some conditions. Training difficulty will be adjusted appropriately following the Challenge Point Framework [49], and adjustments to the training variables such as the force variability and magnitude will be made to keep the participant actively engaged in the intervention. Wheelchair users with no ability to stand or walk will not perform training in standing or walking but will explore other training variations more.

#### Strategies to improve adherence to interventions {11c}

The intervention requires an in-person visit to the laboratory. Support for parking and transportation assistance will be provided as necessary. The visit schedule will be printed and handed out to each participant. Reminder phone calls will be made. A waiting room will be provided within the building for caregivers. Remuneration for participation will be provided. All COVID-19 precautions are taken to ensure the health and safety of participants and study personnel. All training activities will be automatically logged by the custom-developed computer program used for training. The time lapsed for each training session is also provided by the computer program.

#### Relevant concomitant care permitted or prohibited during the trial {11d}

Concurrent upper limb rehabilitation is prohibited during participation in the study. Change in spasticity medication or botulinum toxin injection in the upper limb is prohibited during the study. Concomitant care for other issues is permitted.

#### Provisions for post-trial care {30}

Participants will be followed up until 1 month post-intervention for adverse events. Necessary medical treatment will be provided by the VA.

### Outcomes {12}

The primary outcome measure is the hand function measured by the Action Research Arm Test (ARAT) [50]. The primary time point is from the baseline immediately prior to the intervention to the end of the intervention. Other outcome measures are as follows.

Hand function will also be assessed using the Box and Block Test (BBT) [51]. Patient-centered outcome measures of the Stroke Impact Scale hand and activities of daily living sections and perceived meaningfulness of the intervention [52] will also be obtained. Qualitative interviews will delineate why and how subjects perceive the intervention to be meaningful or not meaningful, after completion of training.

To provide insights into the biomechanical mechanisms underlying the change in hand function, the digit force direction control and muscular coordination will be assessed. The ability to direct digit force will be quantified as the angular deviation of digit force from the target direction. Coordination of upper limb muscles will be assessed using the metrics of (i) the attainable muscle activation space as assessed by electromyography (EMG) workspace volume [53], (ii) motor complexity assessed by the number of paretic muscle synergies [54, 55], (iii) assimilation of the paretic muscle synergy structure toward that of the nonparetic side, and (iii) abnormal flexion synergy [35].

### Participant timeline {13}

**Fig. 4:**
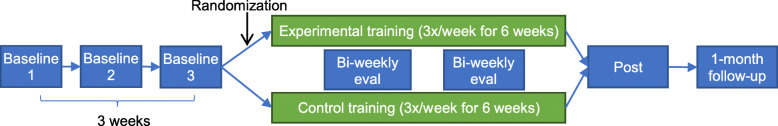
Participation timeline. All participants will have 3 baseline assessments, 6 weeks of training with biweekly assessments, post-assessment, and 1-month follow-up assessment

The participant timeline is shown in Fig. 4. Baseline assessments will take place 3 times over 3 weeks to establish baseline trends. Interventions will entail a total of 18 training sessions over 6 weeks. Biweekly assessments will be performed during a 6-week intervention to examine the pattern of progress. The post-assessment will take place within a week from the last intervention session. The purpose of the 1-month follow-up is to assess retention.

### Sample size {14}

The study was designed primarily to ensure adequate power to analyze the hypothesis on digit force direction. Given the sample size for this hypothesis, detectable effect sizes for the other clinical outcomes were determined. Reduction of force deviation to a value below 20° is expected to result in increased ability to manipulate objects [21]. The mean force deviation for stroke survivors in Chedoke Hand Stage 2–4 was 25.6° [21]. Therefore, a 6° change in force direction is expected to lead to a substantial functional improvement and is considered to be a clinically meaningful difference in force direction. Our pilot data indicate that such change is achievable in stroke survivors with severe hand impairment [30].

Our study is longitudinal with 4 primary time points. In the analyses, an autoregressive (AR(1)) covariance structure will be considered for the within-subject correlations (while other structures will also be examined). For a standard deviation of 6.6° (based on the force direction data in our preliminary study), an alpha level of 0.05, 90% power, and the AR(1) correlation between observations on the same subject of 0.8 (based on the preliminary study), a sample size of 22 participants per group will be adequate to detect a difference of 6°. Adjusting for an expected attrition rate of 15% and screen failure rate of 12%, a sample of 30 per group (for a total of *n* = 60) is planned.

For other outcomes, this sample size will guarantee at least 80% power for respective minimum clinically important differences. For instance, this sample size will be sufficient to detect a change in the ARAT score of 5.7 [50] and BBT score of 5.5 [56] with a power of 80% and 99%, using the standard deviation of 8.1 and 4.3, respectively (based on our preliminary study [30]) for an overall significance of 5%.

### Recruitment {15}

Participants will be recruited from the Ralph H. Johnson VA Medical Center, Charleston, SC. The Charleston VA treats > 300 new stroke cases every year. We will send a recruitment letter to patients who had a stroke identified by the VA Informatics and Computing Infrastructure. We will also receive referrals from clinicians in the outpatient neuro clinic at the VA. A dedicated recruiter will visit the clinic onsite to recruit referred patients. We also have access to the Medical University of South Carolina (MUSC) Stroke Center, a tertiary stroke center that treats > 500 new stroke cases each year. The Institutional Review Board (IRB)-approved MUSC stroke registry has > 1000 stroke survivors who are interested in participating in research. Approximately two-thirds of those in the registries have moderate to severe upper limb impairment appropriate for the trial. The registry continues to grow with around 10 new enrollees each month due to community outreach efforts. These efforts include visiting local stroke support group meetings, organizing stroke caregiver summits and stroke recovery community engagement events, and sending newsletters to develop grassroots connections with stroke survivors, caregivers, clinicians, and clinics in the community. In addition, trial information will be available via the Internet (e.g., ClinicalTrials.gov, South Carolina Research Studies Directory).

### Assignment of interventions: allocation

#### Sequence generation {16a}

A computer-generated random allocation sequence will be used. Block randomization will be used to ensure balance (half in the experimental, half in the control). Block sizes will be random (4, 6 or 8). The block randomization will be stratified by the moderate vs. severe impairment level according to the Fugl-Meyer Assessment of Motor Recovery after Stroke for the Upper Extremity (FMUE) scores [57]. The block randomization will also be stratified equally by sex as a biological variable.

#### Concealment mechanism {16b}

On the first intervention day, the FMUE score and sex will be entered into the custom-developed computer program used for the intervention. The program will then access the computer-generated random sequence for that stratification category, find the next assignment available, and apply that group assignment to the visual feedback display for all training sessions of the participant. Therefore, nobody will know the group assignment until the intervention.

#### Implementation {16c}

The allocation sequence will be generated by the computer. The approved study staff not involved in providing the intervention will enroll the participants. The custom-developed computer program used for training will assign the participants to the group according to the computer-generated allocation sequence.

### Assignment of interventions: blinding

#### Who will be blinded {17a}

Participants will remain naïve to the group assignment as they will not be exposed to the other study condition. Care providers, outcome assessors including qualitative interviewers, and data analysts except for the primary biostatistician will be blinded to the group assignment.

#### Procedure for unblinding if needed {17b}

Permission for unblinding will be deliberated and reviewed by the Data and Safety Monitoring Board (DSMB) if unanticipated intervention-related serious adverse events warrant investigation using the group assignment information.

#### Data collection and management

#### Plans for assessment and collection of outcomes {18a}

The outcome assessment timeline is provided in Fig. 4. All clinical hand function tests (ARAT and BBT) will be administered by a blinded research therapist, videotaped, coded in names, and scored by 2 raters who are blinded to the group assignment as well as the timing of the videos (i.e., before or how many weeks after training). Raters will be trained until *excellent intra/interrater reliability* is met with a correlation greater than 0.9. All force and muscle activity data will be analyzed using a custom-scripted code in MATLAB (the MathWorks, Natick, MA) to obtain the final metrics by the blinded researcher post-data collection.

The clinical hand function tests, ARAT and BBT, have been used to show a significant change with force training in our preliminary study [30]. In addition, both ARAT and BBT have been standardized [58, 59], validated for test-retest and interrater reliability [56, 60], and shown to be responsive to changes in stroke survivors [51]. Clinically meaningful levels of change for ARAT [50] and BBT [56] have been reported. The patient-centered outcome measures of the Stroke Impact Scale [61, 62] hand and activities of daily living sections gauge what participants can do functionally that they could not do before. The perceived meaningfulness of the intervention will be obtained on a 7-point Likert scale [52] (1 = much better; 2 = a little better, meaningful; 3 = a little better, not meaningful; 4 = about the same; 5 = a little worse, not meaningful; 6 = a little worse, meaningful; 7 = much worse) after completion of the training. For qualitative interviews [63], semi-structured interviews with individual participants [64, 65] will be conducted to provide an insightful story about the impact of the training on their life. The key topic will be focused on what constitutes meaningful changes vs. not meaningful, how, and why. The interview will last approximately 30 min. The interviewer will be blinded to the participants’ group assignments.

The digit force direction control is a measure that has been shown to be a powerful biomarker for predicting object grasping abilities and hand function [21]. The previously published methods [21] will be followed for this assessment. Muscular coordination will be assessed using the following well-established metrics. First, the attainable muscle activation space will be computed as EMG workspace *n*-volume representing every recorded EMG vector of activities across upper limb muscles [53]. Second, the degree of motor complexity will be described by the number of paretic muscle synergies [54, 55]. Muscle synergies will be found using the non-negative matrix factorization method [54] and the number of synergies explaining most (> 90%) of the variance in the EMG data [54, 55] will be quantified. Third, assimilation of the paretic muscle synergy structure to the nonparetic side will be quantified as the decreased angle between the subspace defined by the paretic muscle synergies and the subspace defined by the nonparetic muscle synergies [54]. Lastly, to examine training-induced breakaway from the abnormal flexion synergy [35], the extent of co-activation among finger flexor, elbow flexor, and shoulder abductor muscles will be quantified using cross-covariance analysis [66, 67].

#### Plans to promote participant retention and complete follow-up {18b}

To promote participant retention and complete follow-up, effective communication will be maintained between the study staff and participants. Schedules, changes to schedules, and expectation for each visit will be clearly communicated.

#### Data management {19}

All electronic data will be stored in a password-protected secure research server. Visit records in paper will be scanned and stored in the password-protected secure research server. Data will be entered into a computer-based database. Quarterly data quality assessments will be performed by examining the outcomes databases for missing data, unexpected distributions or responses, irregularities, and outliers. Accuracy and completeness of the data collected will also be ensured.

#### Confidentiality {27}

The consent and HIPAA forms where personally identifiable information is recorded will be stored in a locked cabinet in a locked office. Only study personnel will have access to this personally identifiable information. For the video recording of the upper limb function tests, we will set the camera angle such that the video recording does not capture the participant’s face while capturing the hand and arm movements and the interaction between the hand and objects in hand manipulation. For the audio recording of the qualitative interviews, we will not use the participant’s name and will not disclose any identifiable information in the recording, such that the entire audio recording is de-identified. All data will be coded with a participant code, and no personally identifiable information will be used to label the data. This means individual results would not be able to be linked to the participant by others who review the results of this research. De-identified paper data including testing sheets documenting testing sequences and notes will be stored in a cabinet in a key-locked room that is accessible to study personnel only. The linkage between the participant identities and participant codes will be stored in a locked cabinet in a locked room and will be accessible to study personnel only.

#### Plans for collection, laboratory evaluation, and storage of biological specimens for genetic or molecular analysis in this trial/future use {33}

This trial does not involve collecting biological specimens for storage.

## Statistical methods

### Statistical methods for primary and secondary outcomes {20a}

The primary formal analysis for the primary outcome measure will be a repeated measures general linear model with an AR(1) structure, although other structures will be considered and compared. Diagnostics will be performed on the residuals, and appropriate actions will be taken if assumptions required for the statistical tests are not met. The primary independent variables are group (experimental vs. control), evaluation time (3rd baseline, 2 and 4 weeks of training, and post for the primary analyses; all 3 baselines and follow-up will be included in subsequent analyses), and their interaction (group × evaluation time). In addition, we will include sex as an independent variable along with its interactions to study sex differences. If the group × evaluation time interaction is significant, then the main alternative hypothesis of interest, that at post, there is a difference between the two groups, will be tested using post hoc tests. Greater improvement for the experimental than for the control group will support the hypothesis.

In the secondary analysis with 3 baseline measures, the slope of improvement during the baseline will be quantified and tested to determine if it differs from zero, and change in the slope of improvement during training will be compared between the experimental vs. control group. The persistence of the effect will also be examined using data obtained in the follow-up phase of the study.

The same analysis approach will be applied to each of the other outcome measures. Bonferroni correction will be applied to adjust for multiple comparisons. To gauge the level of efficacy of the force training, we will compare the extent of improvements in ARAT, BBT, and Stroke Impact Scale with minimal clinically important differences established in the literature [50, 56, 61, 68] as well as other large trials [68–72]. We will also examine the proportion of subjects who perceive that the intervention has made a meaningful improvement in their function.

### Interim analyses {21b}

No interim analysis is planned. The DSMB may recommend stopping the study if the study has unanticipated safety concerns that warrant stopping.

### Methods for additional analyses (e.g., subgroup analyses) {20b}

Other covariates such as initial impairment levels, stroke type, spasticity, and somatosensory deficits will be included. Given the number of covariates, we will first use univariate analyses to choose a smaller pool of potential predictors and apply model selection methods such as the forward stepwise regression approach. Multivariate extensions that include all variables together will be considered.

Correlations between outcome measures will be calculated. Data reduction methods, such as factor analysis or principal component analysis, will be considered if the multiple outcome measures are highly correlated.

### Methods in analysis to handle protocol non-adherence and any statistical methods to handle missing data {20c}

We will use intent-to-treat analysis. If missing data arise, multiple imputation methods will be applied under the assumption of missing at random.

### Plans to give access to the full protocol, participant-level data, and statistical code {31c}

The protocol will be shared on ClinicalTrials.gov. De-identified participant-level dataset and/or statistical code will be shared upon reasonable request in writing.

### Oversight and monitoring

#### Composition of the coordinating center and trial steering committee {5d}

Study oversight will be provided by the DSMB. The DSMB will be composed of a board-certified stroke neurologist, a registered and licensed occupational therapist, and a biostatistician with expertise in the design and analysis of clinical trials. The DSMB members will be experienced in the care of stroke survivors and/or stroke recovery research. The DSMB will convene semiannually to review the enrollment and study progression.

The trial management will be performed by the principal investigator, co-investigators, and the IRB-approved study personnel. The trial team will meet weekly or as necessary to discuss the trial setup, operation, progression, data analysis, interpretation, and dissemination. The principal investigator and study personnel are responsible for the day-to-day operation and organization of the trial including identifying potential recruits and taking consent.

#### Composition of the data monitoring committee, its role, and reporting structure {21a}

The DSMB will also ensure the safety of participants and the validity and integrity of data collected during the study. The DSMB will review the adverse event data and provide a report to the IRB. The DSMB will be independent from the sponsor and competing interests.

#### Adverse event reporting and harms {22}

Adverse events will be solicited at each visit, recorded, and coded in terms of frequency, severity, relatedness to the intervention, and unanticipated nature using established guidelines [73–75]. All serious adverse events will be investigated by an independent medical monitor to determine the relatedness to the intervention. The report by the independent medical monitor will be reviewed by the DSMB. All related serious adverse events will be reported to the IRB as they occur. All adverse event data will be tabulated and reported to the DSMB and ClinicalTrials.gov.

#### Frequency and plans for auditing trial conduct {23}

The sponsor will audit the study annually. The sponsor will review the study progression, regulatory compliance, and training compliance of all study personnel.

#### Plans for communicating important protocol amendments to relevant parties (e.g., trial participants, ethical committees) {25}

Any changes will be approved by the IRB prior to being in effect. Changes will be updated in ClinicalTrials.gov.

#### Dissemination plans {31a}

Trial results will be disseminated in ClinicalTrials.gov, in publications, and in conferences and in-service/community presentations.

## Discussion

To ensure participants understand the task, participants may perform the task with the unaffected hand and demonstrate their understanding of the training prior to training with the affected hand. For participants with a high level of tonic muscle tone in the finger flexor that interferes with producing force in the extension direction, the resting-state grip force may be zeroed out to facilitate the training. While primary statistical analysis is focused on determining the group and evaluation time interaction effect, the association between the changes in upper limb function, digit force direction, and muscle activation will be examined using correlations.

This trial will evaluate a novel training tool to improve force control, thereby addressing the unmet need in the current rehabilitation. This research will also determine the underlying muscular mechanisms as well as the training’s impact on the functional use of the hand. As a result, this research is expected to enhance hand function, contributing to improved independence and quality of life post-stroke.

## Trial status

Protocol #9. February 8, 2022. Recruitment began on October 28, 2020, and is expected to conclude in October 2023.

## Supplementary Information

**Additional file 1.**

## Abbreviations

ARAT: Action Research Arm Test
AR(1): Autoregressive
BBT: Box and Block Test
DSMB: Data and Safety Monitoring Board
EMG: Electromyography
FMUE: Fugl-Meyer Assessment of Motor Recovery after Stroke for the Upper Extremity
HIPAA: Health Insurance Portability and Accountability Act
IRB: Institutional Review Board
MUSC: Medical University of South Carolina
NIH: National Institutes of Health
SC: South Carolina
USA: United States of America
VA: Veterans Affairs
