# Supplementary material for: Effect of novel training to normalize altered finger force direction post-stroke: study protocol for a double-blind randomized controlled trial
Source: Trials. 2022 Apr 12;23:301. doi: 10.1186/s13063-022-06224-w (PMC9003156; doi:10.1186/s13063-022-06224-w)
Supplement: Supplementary file 1 — Additional file 1. [file 13063_2022_6224_MOESM1_ESM.pdf]

SPRINGER NATURE

Change of authorship request form - Journals (pre-acceptance)

Important information. Please read.

- This form should be used by authors to request any change in authorship (adding/deleting authors) including changes in corresponding authors. This form should not be used for name changes. Please fully complete all sections. Use black ink and block capitals and provide each author's full name with the given name first followed by the family name.

- By signing this declaration, all authors guarantee that the order of the authors are in accordance with their scientific contribution, if applicable as different conventions apply per discipline, and that only authors have been added who made a meaningful contribution to the work.

- Please note, in author collaborations where there is formal agreement for representing the collaboration, it is sufficient for the representative or legal guarantor (usually the corresponding author) to complete and sign the Authorship Change Form on behalf of all authors, **next to the added/removed author(s)**. **(Complete Section 3, followed by Section 6.)**
  In author collaborations where there is no formal agreement for representing the collaboration and **there are more than 10 authors**, one may sign for all, provided the signer appends correspondence that attests that each of the authors have agreed to the change **and the added/removed authors sign the form**. **(Complete Section 3, followed by Section 6.)**

- Please note, we cannot investigate or mediate any authorship disputes. If you are unable to obtain agreement from all authors (including those who you wish to be removed) you must refer the matter to your institution(s) for investigation. Please inform us if you need to do this.

- If you are not able to return a fully completed form within **30 days** of the date that it was sent to the author requesting the change, we may have to withdraw your manuscript. We cannot publish manuscripts where authorship has not been agreed by all authors (including those who have been removed).

- Incomplete forms will be rejected.

- Please return/upload this form, fully completed, to the Journals Editorial Office. The Journal and/or Publisher will consider the information you have provided to decide whether to approve the proposed change in authorship. We may decide to contact your institution for more information or undertake a further investigation, if appropriate, before making a final decision.

SPRINGER NATURE Springer Nature is one of the world's leading global research, educational and professional publishers, created in May 2015 through the combination of Nature Publishing Group, Palgrave Macmillan, Macmillan Education and Springer Science+Business Media.

SPRINGER NATURE

Change of authorship request form - Journals (pre-acceptance)

Section 1: Please provide the current title of manuscript

Manuscript ID no.: TRLS-D-22-00047

Title: **Effect of novel training to normalize altered finger force direction post stroke: study protocol for a double-blind randomized controlled trial**

Section 2: Please provide the previous authorship, in the order shown on the manuscript before the changes were introduced. Please indicate the corresponding author by adding (CA) behind the name.

	First name(s)	Family name	ORCID or SCOPUS id, if available
1st author	Na Jin	Seo	0000-0001-6446-5905
2nd author	Derek	Kamper	0000-0001-9048-4106
3rd author	Viswanathan	Ramakrishnan	0000-0002-4098-0539
4th author	Jillian	Harvey	0000-0002-6814-8226
5th author	Christian	Finetto	0000-0003-0520-2034
6th author	Christian	Schranz	0000-0003-1102-7180
7th author	Gabrielle	Scronce	0000-0002-3861-1371
8th author	Kristen	Coupland	
9th author	Keith	Howard	
10th author	Jenna	Blaschke	

Please use an additional sheet if there are more than 10 authors.

11th author	Adam	Baker
12th author	Caitlyn	Meinzer
13th author	Robert	Adams

SPRINGER NATURE Springer Nature is one of the world's leading global research, educational and professional publishers, created in May 2015 through the combination of Nature Publishing Group, Palgrave Macmillan, Macmillan Education and Springer Science+Business Media.

SPRINGER NATURE

Change of authorship request form - Journals (pre-acceptance)

Section 3: Please provide a justification for change. Please use this section to explain your reasons for changing the authorship of your manuscript, e.g. what necessitated the change in authorship? Please refer to the (journal) policy pages for more information about authorship. Please explain why omitted authors were not originally included and/or why authors were removed on the submitted manuscript.

Dr. Craig Velozo is a member of the Data and Safety Monitoring Board for the trial and has substantially contributed to the trial design and monitoring. He did not have time to review the manuscript at the time of submission. Since then, he has reviewed the manuscript, provided substantial comments, approved the manuscript, and agreed to be accountable as an author and ensure integrity of the work.

Section 4: Proposed new authorship. Please provide your new authorship list in the order you would like it to appear on the manuscript. Please indicate the corresponding author by adding (CA) behind the name. If the Corresponding Author has changed, please indicate the reason under section 3.

	First name(s)	Family name (this name will appear in full on the final publication and will be searchable in various abstract and indexing databases)	Affiliated institute	E-mail address
1st author	Na Jin	Seo (CA)	Medical University of South Carolina	seon@musc.edu
2nd author	Derek	Kamper	North Carolina State University	dgkamper@ncsu.edu
3rd author	Viswanathan	Ramakrishnan	Medical University of South Carolina	ramakris@musc.edu
4th author	Jillian	Harvey	Medical University of South Carolina	harveyji@musc.edu
5th author	Christian	Finetto	Medical University of South Carolina	finetto@musc.edu
6th author	Christian	Schranz	Medical University of South Carolina	schranz@musc.edu
7th author	Gabrielle	Scronce	Medical University of South Carolina	scronce@musc.edu
8th author	Kristen	Coupland	Medical University of South Carolina	coupland@musc.edu
9th author	Keith	Howard	Medical University of South Carolina	howardke@musc.edu
10th author	Jenna	Blaschke	Medical University of South Carolina	blaschkj@musc.edu
11th author	Adam	Baker	Medical University of South Carolina	bakerdon@musc.edu
12th author	Caitlyn	Meinzer	Medical University of South Carolina	ellerbcn@musc.edu
13th author	Craig	Velozo	Medical University of South Carolina	velozo@musc.edu
14th author	Robert	Adams	Medical University of South Carolina	adamsrj@musc.edu

SPRINGER NATURE Springer Nature is one of the world's leading global research, educational and professional publishers, created in May 2015 through the combination of Nature Publishing Group, Palgrave Macmillan, Macmillan Education and Springer Science+Business Media.

Change of authorship request form - Journals (pre-acceptance)

Section 5: Author contribution, Acknowledgement and Disclosures. Please use this section to provide a new disclosure statement and, if appropriate, acknowledge any contributors who have been removed as authors and ensure you state what contribution any new authors made (if applicable per the journal or book (series) policy). Please ensure these are updated in your manuscript - after approval of the change(s) - as our production department will not transfer the information in this form to your manuscript.

New acknowledgements:

Dr. Velozo will be removed from the acknowledgment as he is now an author.

New Disclosures (financial and non-financial interests, funding):

N/A

New Author Contributions statement (if applicable per the journal policy):

CM, CV, and RA serve in the Data and Safety Monitoring Board.
CV is the added author, Dr. Velozo.

State 'Not applicable' if there are no new authors.

SPRINGER NATURE Springer Nature is one of the world's leading global research, educational and professional publishers, created in May 2015 through the combination of Nature Publishing Group, Palgrave Macmillan, Macmillan Education and Springer Science+Business Media.

SPRINGER NATURE

Change of authorship request form - Journals (pre-acceptance)

Section 6: Declaration of agreement. *All* authors, unchanged, new and removed *must* sign this declaration.
(NB: Please print the form, (docu)-sign and return/upload a scanned copy. Please note that signatures that have been inserted as an image file are acceptable as long as it is handwritten. Typed names in the signature box are unacceptable.) *** Please delete as appropriate. Delete all of the bold if you were on the original authorship list and are remaining as an author.**

	First name	Family name		Signature	Date
1st author	**Na Jin**	**Seo**	I agree to the proposed new authorship shown in section 4 ~~**/and the addition/removal*of my name to the authorship list**~~ /and the proposed change in corresponding author	[illegible]	2/11/2022
2nd author	**Derek**	**Kamper**	I agree to the proposed new authorship shown in section 4 ~~**/and the addition/removal*of my name to the authorship list**~~ /and the proposed change in corresponding author		
3rd author	**Viswanathan**	**Ramakrishnan**	I agree to the proposed new authorship shown in section 4 ~~**/and the addition/removal*of my name to the authorship list**~~ /and the proposed change in corresponding author	[illegible]	2/16/22
4th authors	**Jillian**	**Harvey**	I agree to the proposed new authorship shown in section 4 ~~**/and the addition/removal*of my name to the authorship list**~~ /and the proposed change in corresponding author		
5th author	**Christian**	**Finetto**	I agree to the proposed new authorship shown in section 4 ~~**/and the addition/removal*of my name to the authorship list**~~ /and the proposed change in corresponding author		
6th author	**Christian**	**Schranz**	I agree to the proposed new authorship shown in section 4 ~~**/and the addition/removal*of my name to the authorship list**~~ /and the proposed change in corresponding author	[illegible]	2/16/22
7th author	**Gabrielle**	**Scronce**	I agree to the proposed new authorship shown in section 4 ~~**/and the addition/removal*of my name to the authorship list**~~ /and the proposed change in corresponding author	[illegible]	2/16/22

SPRINGER NATURE Springer Nature is one of the world's leading global research, educational and professional publishers, created in May 2015 through the combination of Nature Publishing Group, Palgrave Macmillan, Macmillan Education and Springer Science+Business Media.

SPRINGER NATURE

Change of authorship request form - Journals (pre-acceptance)

	First name	Family name		Signature	Date
8th author	**Kristen**	**Coupland**	I agree to the proposed new authorship shown in section 4 ~~/and the addition/removal*of my name to the authorship list~~ /and the proposed change in corresponding author		2/16/2022
9th author	**Keith**	**Howard**	I agree to the proposed new authorship shown in section 4 ~~/and the addition/removal*of my name to the authorship list~~ /and the proposed change in corresponding author		2/16/2022
10th author	**Jenna**	**Blaschke**	I agree to the proposed new authorship shown in section 4 ~~/and the addition/removal*of my name to the authorship list~~ /and the proposed change in corresponding author		

Please use an additional sheet if there are more than 10 authors.

In case of author collaborations with formal agreement:

	Name of consortium/consortia	First name	Family name		Signature	Date
Representative/legal guarantor				I agree to the proposed new authorship shown in section 4 /**and the addition/removal*of my name to the authorship list** /and the proposed change in corresponding author		

Both added/removed authors should complete the information in the first table under Section 6.

Please see the next page for more authors.

---- End of form ----

SPRINGER NATURE Springer Nature is one of the world's leading global research, educational and professional publishers, created in May 2015 through the combination of Nature Publishing Group, Palgrave Macmillan, Macmillan Education and Springer Science+Business Media.

SPRINGER NATURE Change of authorship request form - Journals (pre-acceptance)

Section 6: Declaration of agreement. *All* authors, unchanged, new and removed *must* sign this declaration.
(NB: Please print the form, (docu)-sign and return/upload a scanned copy. Please note that signatures that have been inserted as an image file are acceptable as long as it is handwritten. Typed names in the signature box are unacceptable.) *** Please delete as appropriate. Delete all of the bold if you were on the original authorship list and are remaining as an author.**

	First name	Family name		Signature	Date
~~1st author~~ 11th author	**Adam**	**Baker**	I agree to the proposed new authorship shown in section 4 ~~/and the addition/removal*of my name to the authorship list~~ /and the proposed change in corresponding author	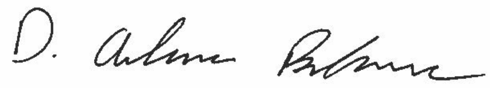	**2/16/22**
~~2nd author~~ 12th author	**Caitlyn**	**Meinzer**	I agree to the proposed new authorship shown in section 4 ~~/and the addition/removal*of my name to the authorship list~~ /and the proposed change in corresponding author		
~~3rd author~~ 13th author	**Craig**	**Velozo**	I agree to the proposed new authorship shown in section 4 **/and the addition/~~removal~~*of my name to the authorship list** /and the proposed change in corresponding author	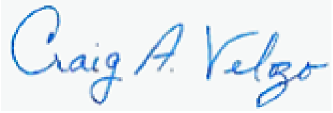	02/11/2022
~~4th authors~~ 14th author	**Robert**	**Adams**	I agree to the proposed new authorship shown in section 4 ~~/and the addition/removal*of my name to the authorship list~~ /and the proposed change in corresponding author		
5th author			I agree to the proposed new authorship shown in section 4 **/and the addition/removal*of my name to the authorship list** /and the proposed change in corresponding author		
6th author			I agree to the proposed new authorship shown in section 4 **/and the addition/removal*of my name to the authorship list** /and the proposed change in corresponding author		
7th author			I agree to the proposed new authorship shown in section 4 **/and the addition/removal*of my name to the authorship list** /and the proposed change in corresponding author		

SPRINGER NATURE Springer Nature is one of the world's leading global research, educational and professional publishers, created in May 2015 through the combination of Nature Publishing Group, Palgrave Macmillan, Macmillan Education and Springer Science+Business Media.

Subject: Re: Your approval needed
Date: Wednesday, February 16, 2022 at 2:16:27 PM Eastern Standard Time
From: Derek Kamper
To: Seo, Na Jin

CAUTION: External

I approve.

Derek

On Wed, Feb 16, 2022 at 1:32 PM Seo, Na Jin <seon@musc.edu> wrote:

> Hi Derek,
>
> This email is regarding the VA trial protocol manuscript that was submitted to the journal, Trials.
>
> Dr. Craig Velozo is a member of the Data and Safety Monitoring Board and has substantially contributed to the trial design and monitoring. He was not included as an author in the original submission because he did not have time to review/approve the manuscript at the time of submission. Since then, he has reviewed the manuscript, provided substantial feedback, approved the manuscript, and agreed to be an author.
>
> Could you please **indicate if you approve** adding Dr. Velozo to the author list for the paper?
>
> Thank you!
>
> Na Jin

--
Derek Kamper, Ph.D.
Associate Professor, Joint Department of Biomedical Engineering
Associate Director, Closed Loop Engineering for Advanced Rehabilitation (CLEAR)
North Carolina State University
University of North Carolina at Chapel Hill
(919) 515-4411

Subject: Re: Your approval needed
Date: Wednesday, February 16, 2022 at 5:56:51 PM Eastern Standard Time
From: Harvey, Jillian
To: Seo, Na Jin

Yes. I approve. Thanks!

Get Outlook for iOS

From: Seo, Na Jin <seon@musc.edu>
Sent: Wednesday, February 16, 2022 12:34:39 PM
To: Harvey, Jillian <harveyji@musc.edu>
Subject: Your approval needed

Hi Jillian,

This email is regarding the VA trial protocol manuscript that was submitted to the journal, Trials.

Dr. Craig Velozo is a member of the Data and Safety Monitoring Board and has substantially contributed to the trial design and monitoring. He was not included as an author in the original submission because he did not have time to review/approve the manuscript at the time of submission. Since then, he has reviewed the manuscript, provided substantial feedback, approved the manuscript, and agreed to be an author.

Could you please **indicate if you approve** adding Dr. Velozo to the author list for the paper?

Thank you!
Na Jin

Subject: Re: Your signature needed
Date: Wednesday, February 16, 2022 at 3:43:59 PM Eastern Standard Time
From: Finetto, Christian
To: Seo, Na Jin

Hi Na Jin,

I certainly approve. Thanks,

Christian

From: Seo, Na Jin <seon@musc.edu>
Sent: Wednesday, February 16, 2022 2:25 PM
To: Finetto, Christian <finetto@musc.edu>; Schranz, Christian <schranz@musc.edu>; Scronce, Gabrielle <scronce@musc.edu>; Howard, Keith <howardke@musc.edu>; Blaschke, Jenna <blaschkj@musc.edu>
Subject: Re: Your signature needed

This email is regarding the VA trial protocol manuscript that was submitted to the journal, Trials.
Dr. Craig Velozo is a member of the Data and Safety Monitoring Board and has substantially contributed to the trial design and monitoring. He was not included as an author in the original submission because he did not have time to review/approve the manuscript at the time of submission. Since then, he has reviewed the manuscript, provided substantial feedback, approved the manuscript, and agreed to be an author.
Could you please reply to me and **indicate if you approve** adding Dr. Velozo to the author list for the paper? You can do that in lieu of signing on the Author Form.
Thank you!
Na Jin

Subject: RE: Your signature needed
Date: Wednesday, February 16, 2022 at 2:31:01 PM Eastern Standard Time
From: Scronce, Gabrielle
To: Seo, Na Jin

I approve.

From: Seo, Na Jin <seon@musc.edu>
Sent: Wednesday, February 16, 2022 2:25 PM
To: Finetto, Christian <finetto@musc.edu>; Schranz, Christian <schranz@musc.edu>; Scronce, Gabrielle <scronce@musc.edu>; Howard, Keith <howardke@musc.edu>; Blaschke, Jenna <blaschkj@musc.edu>
Subject: Re: Your signature needed

This email is regarding the VA trial protocol manuscript that was submitted to the journal, Trials.
Dr. Craig Velozo is a member of the Data and Safety Monitoring Board and has substantially contributed to the trial design and monitoring. He was not included as an author in the original submission because he did not have time to review/approve the manuscript at the time of submission. Since then, he has reviewed the manuscript, provided substantial feedback, approved the manuscript, and agreed to be an author.
Could you please reply to me and **indicate if you approve** adding Dr. Velozo to the author list for the paper? You can do that in lieu of signing on the Author Form.
Thank you!
Na Jin

Subject: Re: Your approval needed
Date: Wednesday, February 16, 2022 at 9:44:22 PM Eastern Standard Time
From: Blaschke, Jenna
To: Seo, Na Jin

Hi Dr. Seo,

I approve adding Dr. Velozo to the author list for the VA trial protocol paper.

All the best,
Jenna

Get Outlook for Android

From: Seo, Na Jin <seon@musc.edu>
Sent: Wednesday, February 16, 2022 9:39:56 PM
To: Blaschke, Jenna <blaschkj@musc.edu>
Subject: Your approval needed

Hi Jenna,

This email is regarding the VA trial protocol manuscript that was submitted to the journal, Trials.

Dr. Craig Velozo is a member of the Data and Safety Monitoring Board and has substantially contributed to the trial design and monitoring. He was not included as an author in the original submission because he did not have time to review/approve the manuscript at the time of submission. Since then, he has reviewed the manuscript, provided substantial feedback, approved the manuscript, and agreed to be an author.

Could you please reply to me and **indicate if you approve** adding Dr. Velozo to the author list for the paper?

Thank you!
Na Jin

Subject: Re: Your approval needed
Date: Wednesday, February 16, 2022 at 1:53:53 PM Eastern Standard Time
From: Meinzer, Caitlyn
To: Seo, Na Jin

I approve, thanks

Get Outlook for Android

From: Seo, Na Jin <seon@musc.edu>
Sent: Wednesday, February 16, 2022 1:35:39 PM
To: Meinzer, Caitlyn <ellerbcn@musc.edu>
Subject: Your approval needed

Hi Caitlyn,

This email is regarding the VA trial protocol manuscript that was submitted to the journal, Trials.

Dr. Craig Velozo is a member of the Data and Safety Monitoring Board and has substantially contributed to the trial design and monitoring. He was not included as an author in the original submission because he did not have time to review/approve the manuscript at the time of submission. Since then, he has reviewed the manuscript, provided substantial feedback, approved the manuscript, and agreed to be an author.

Could you please **indicate if you approve** adding Dr. Velozo to the author list for the paper?

Thank you!
Na Jin

Subject: RE: Your approval needed
Date: Wednesday, February 16, 2022 at 2:16:15 PM Eastern Standard Time
From: Adams, Robert J.
To: Seo, Na Jin

yes if he meets criteria of course he should be added. Robert

Robert J Adams MS MD
Distinguished Professor of Neurology
Director South Carolina Stroke Center of Economic Excellence
Department of Neurology
Medical University South Carolina
Charleston, SC 29425
President Zeriscope, Inc

From: Seo, Na Jin <seon@musc.edu>
Sent: Wednesday, February 16, 2022 1:35 PM
To: Adams, Robert J. <adamsrj@musc.edu>
Subject: Your approval needed

Hi Dr. Adams,

This email is regarding the VA trial protocol manuscript that was submitted to the journal, Trials.

Dr. Craig Velozo is a member of the Data and Safety Monitoring Board and has substantially contributed to the trial design and monitoring. He was not included as an author in the original submission because he did not have time to review/approve the manuscript at the time of submission. Since then, he has reviewed the manuscript, provided substantial feedback, approved the manuscript, and agreed to be an author.

Could you please **indicate if you approve** adding Dr. Velozo to the author list for the paper?

Thank you!
Na Jin
